# Book review: Strategies for radiation therapy treatment planning

**DOI:** 10.1002/acm2.12602

**Published:** 2019-05-14

**Authors:** Chengyu Shi

**Affiliations:** ^1^ Memorial Sloan Kettering Cancer Center Middletown NJ USA

## 1 INTRODUCTION

Radiation therapy is one of the most effective methods for cancer treatment. During radiation therapy, treatment planning is crucial for successfully treating the diseases while sparing to the furthest extent the healthy tissues. A recently published book entitled “Strategies for Radiation Therapy Treatment Planning” provides readers with a highly serviceable resource about the technical aspects of radiotherapy treatment planning.

## 2 PURPOSE AND AUDIENCE

As mentioned by the senior editor, Dr. Ping Xia, in the preface, the birth of this book was inspired by her colleague, Dr. Gregory Videtic, who is a senior author of “Handbook of Treatment Planning in Radiation Oncology”. Comparing with earlier published books about radiotherapy treatment planning, which are prone to the pedagogical side as textbooks, this new book serves an unmet need as a pocket‐sized book with details and up‐to‐date information for user’s quick resource for treatment planning knowledge. Therefore, the book is designed as a convenient instructional handbook. This book also serves as a companion to Videtic’s book that focuses on the audiences of radiation oncologists. The current book is more toward the interests of medical dosimetrists and medical physicists, while also serving well as a treatment planning resource book that goes well with Videtic’s book for radiation oncologists and other potential audiences who want to get familiar with radiation therapy practice.

## 3 CONTENTS AND HIGHLIGHTS

The book has 14 chapters and about 300 pages. The first three chapters are general overviews about treatment planning: The principles of external beam treatment planning; inverse planning and advanced treatment planning tools; simulation and verification. Chapters four to 14 cover detailed treatment planning strategies for each treatment site ‐ central nervous system, head and neck planning, breast cancer, thoracic cancer, gastrointestinal radiotherapy, genitourinary cancer, gynecologic cancer, lymphoma, soft tissue sarcoma, pediatric cancer, and palliative treatment. In each site‐specific chapter, it covers from simulation, the general principle for planning, case representation, to specific techniques. It also discusses detailed key points in the simulation, how the target and organ at risks are defined, the planning objectives, the planning technique, as well as the expected outcome of the treatment planning. Compared to other general treatment planning books, the highlights of this book are the typical examples, illustrations, tabulated elaborate metrics for dosimetry, completeness of treatment sites.

## 4 CRITICAL ASSESSMENT

This book focuses on the technical aspects of treatment planning and targets on practical implementation. Besides the treatment planning details, archetypal examples, informative illustrations, and tables, the book represents the teamwork of radiation oncologists, medical physicists, medical dosimetrists, and radiation therapists of Cleveland Clinic. The teamed authors shared their invaluable experiences with immobilization devices, modality choices, site specific planning strategies, and evaluation metrics to the audiences.

The book also covers topics beyond the routine strategies of treatment planning. Leading edge techniques and special cases, such as auto planning, knowledge‐based planning, adaptive planning for head and neck patients, motion management strategies, difficult and challenging re‐irritation cases, are presented for readers who seek a potential solution for their own needs from the book.

The book is mostly on external beam radiation therapy planning. However, it also covers a few topics about brachytherapy planning, such as low dose rate prostate seed implant and gynecologic high dose rate brachytherapy planning. In the early chapters, the authors prepared the readers with brief but deep enough review about inverse treatment planning, modern technology for intensity modulated radiation therapy and volumetric modulated arc therapy. This will benefit readers with less experience in this field.

The book is an excellent summary of the treatment planning experiences of the radiation oncology team at Cleveland Clinic with their specific treatment planning systems. The readers using different planning systems could easily adapt the experiences in the book into their own clinic practice.

In summary, “Strategies for Radiation Therapy Treatment Planning” is a handy and essential reference for modern treatment planning. It is therefore recommended as a valuable book for the bookshelf and pocket of everyone involved in radiotherapy treatment planning.

## 5 ABOUT THE REVIEWER

Dr. Chengyu Shi has been working in therapeutic physics field for over 14 years. He has taught medical dosimetrist and medical physicists on treatment planning. He is an American Board of Radiology certified medical physicist and ASTRO APEx Surveyor. He is currently a lead medical physicist at the Memorial Sloan Kettering Cancer Center at Basking Ridge and Monmouth campuses in New Jersey.

